# Language and Beyond: A Registered Report Examining Single and Multiple Risk Models of Later Reading Comprehension Weaknesses

**DOI:** 10.1111/desc.70048

**Published:** 2025-07-14

**Authors:** Emma James, Paul A. Thompson, Lucy Bowes, Kate Nation

**Affiliations:** ^1^ Department of Experimental Psychology University of Oxford Oxford UK; ^2^ Department of Psychology University of York York UK; ^3^ Centre for Educational Development, Appraisal and Research University of Warwick Coventry UK

**Keywords:** reading comprehension, poor comprehenders, early language, multiple risk, ALSPAC

## Abstract

Children with poor reading comprehension tend to have oral language weaknesses, suggesting that poor language in the early years is a proximal cause of later reading comprehension difficulties. Yet, longitudinal studies have not succeeded in reliably predicting which children go on to have comprehension weaknesses (CW), and evidence comprises small sample sizes and a narrow focus on language in isolation. In this registered report, we examined early predictors of later outcomes in 879 children with CW identified at 9 years in the Avon Longitudinal Study of Parents and Children. Study 1 showed that these children had poorer language at 24 and 38 months (but not 15 months) than peers who did not go on to develop reading difficulties (*n* = 4516). However, preschool language ability was a poor predictor of individual outcomes, which was not improved by accounting for the language and communication environment. Study 2 used a multiple risk approach to ask whether breadth (number) or depth (severity) of risk factors predicted reading outcomes in a subsample of 125 children with CW and 561 typically developing readers. Having three or more risk factors increases the risk for later CW. Language was the most consistent predictor of group membership, but the depth of cognitive risks beyond language was associated with the severity of reading impairment. However, neither the breadth nor the depth of risks could adequately predict individual outcomes. These findings align with a multiple risk view of reading CW and highlight the challenges in early identification.

**Summary:**

- Children with comprehension weaknesses (CW) in mid‐childhood had language weaknesses at 24 and 38 months, but prediction was poor at an individual child level.
- The breadth of early risk factors related to both the presence and severity of later CW, but remained poor predictors of individual outcomes.
- While lower levels of preschool language were a risk factor for poor comprehension, other cognitive factors were associated with the severity of difficulties.
- These findings highlight the challenge in identifying children at risk for comprehension difficulties, and emphasise the need to look both at language and beyond language.

## 1 Introduction

Children who start school with low levels of oral language ability are at considerable risk for later reading difficulties (Snowling and Hulme 2021). However, both language and reading are complex and multi‐faceted traits, and questions remain about who is at risk and why. School‐aged children with dyslexia find it difficult to identify and read words, and there is substantial evidence pointing to a range of speech and language difficulties in the pre‐school years that confer risk for later reading difficulties (Snowling and Melby‐Lervåg 2016). Our focus is with a different group of children whose reading difficulties lie in comprehension, often referred to as ‘poor comprehenders’^1^ (Cain 2022; Hulme and Snowling 2011). While school‐age children with comprehension weaknesses (CW) have oral language weaknesses that may underpin their difficulties with reading (Catts et al. 2006), the relationship between early language and reading outcomes in children later identified as having CW is not well understood. In this registered report, Study 1 examined whether early language weaknesses could be identified at 15‐, 24‐ and 36‐months of age in a large sample of children (*n* = 879) identified as having CW at 9 years of age. We examined the extent to which early language ability and the broader communicative context in which it occurs can predict later comprehension outcomes and, crucially, whether the two factors interact to exacerbate risk. The availability of additional data for a subsample of these children (*n* = 125) allowed Study 2 to advance this multiple risk approach, building on work by Hayiou‐Thomas et al. (2021) who found that language and literacy outcomes in adolescence were predicted by the breadth of preschool difficulties (i.e., number of risk factors), with little to be gained by information on severity. Our analyses tested whether this conclusion extends to those children who show weaker comprehension than decoding. Together, the findings of Studies 1 and 2 will have important implications for identifying risk for later comprehension impairments, and for the type and timing of effective interventions.

### 1.1 The Early Language Skills of Children with Comprehension Weaknesses

‘Poor comprehenders’ have typically been identified in mid‐childhood as children who have poor reading comprehension despite age‐appropriate word reading and decoding ability (Keenan et al. 2014; Nation and Snowling, 1997). These children typically underperform relative to typically developing (TD) peers on measures of vocabulary, grammar and higher‐level comprehension (Cain et al. 2001; Catts et al. 2006; Nation et al. 2004; Oakhill et al. 2005). These language weaknesses are already observable by the time that children start school (Catts et al. 2006; Elwér et al. 2015; Nation et al. 2010; Torppa et al. 2007), indicating that they are not simply a consequence of poor reading comprehension itself. This observation holds promise for signposting early intervention: if additional support can be provided before children begin to read, then more widespread academic difficulties may be avoidable (Torppa et al. 2020). Importantly, however, this relies on identifying risk reliably and validly. Some initial findings were reported by Justice et al. (2013), who used longitudinal data to ask how early in development language weaknesses could be observed in 16 children who were later identified as having CW in fifth grade. They found that these children had poorer language comprehension scores at 36 and 54 months of age, which were statistically different from children who went on to become typical readers. Medium effect sizes were also observable at 15 and 24 months, but these were not statistically robust. However, as noted by the authors, the ability to detect group differences was severely limited by the small sample size (*n* = 16). Further investigation is clearly needed in a sample large enough to quantify group differences at each age.

Group differences were quite large at 54 months, but Justice et al. noted that performance on these preschool language measures was a poor predictor of later group membership. Although this conclusion is likely affected by the small sample, it does align with other studies that show modest correlations between preschool language and school‐aged literacy alongside insufficient precision to identify individual risk (e.g., Elwér et al. 2013; Duff, Reen et al. 2015). In this registered report, we consider three factors that likely contribute to this poor predictive ability. First, the categorisation of later reading comprehension difficulties often relies upon stringent yet arbitrary selection criteria, leaving little to dissociate between children who fall on either side of the thresholds imposed. Second, there is substantial variability in the growth of language skills across the preschool years, leaving scope for children to catch up with their peers after the language assessment (e.g., Dale et al. 2003; Henrichs et al. 2011) and prior to learning to read—an important milestone for considering long‐lasting literacy difficulties (Stothard et al. 1998). We know that there are substantial environmental influences on early language delay (Bishop et al. 2003), and that measures of the home environment can predict growth in early language (Sénéchal and LeFevre 2014; Son and Morrison 2010). Thus, early language difficulties within a rich communicative environment may be less likely to have long‐term consequences than those in more impoverished environments. Third, reading comprehension is the product of many language and cognitive skills (Castles et al. 2018), such that variations across a range of cognitive domains may influence reading outcomes on an individual level. Together, these indicate that we need to look beyond language to gain a better understanding of who is at risk for reading comprehension difficulties.

### 1.2 Comprehension Weaknesses Within a Multiple Risk Framework

According to multiple risk models (e.g., Pennington 2006; Astle and Fletcher‐Watson 2020; Catts and Petscher 2022), developmental disorders arise due to multiple risk (and resilience) factors that are each probabilistically associated with outcomes rather than a single core deficit. The impression of a core deficit is a likely consequence of using restrictive selection criteria in case‐control research designs, neglecting the heterogeneity of difficulties that exist within the population (Astle and Fletcher‐Watson 2020). To consider children with CW within this framework, while language difficulties are most robustly identified on a group level, it is possible that less severe or more varied problems have gone undetected in previous studies that are characterised by small case‐control samples and a restricted range of measures. This suggestion aligns with the observation from a meta‐analysis that poor language alone may be insufficient in accounting for the severity of reading CW (Spencer et al. 2019), and that a range of cognitive weaknesses accompany reading difficulties across development (Peng et al. 2022).

Beyond language and environmental risk, we consider three aspects of broader cognition in our analyses of children with CW, namely nonverbal ability, working memory and phonological skills. When it is not controlled for in group selection, children with CW tend to show lower nonverbal IQ than peers indicating that there is variability that might be associated with reading outcomes (e.g., Nation et al. 2002; Catts et al. 2006; James et al. 2023), but it is not known whether it represents an early risk factor. Reading comprehension places demands on memory and executive skills, and there is some evidence that children with CW show poor working memory, especially when measured using complex verbal tasks (Carretti et al. 2009). Whether working memory deficits in early childhood are associated with later risk for later CW is also not known. Finally, although school‐aged children with CW do not tend to show phonological weaknesses (e.g., Cain et al. 2000; Nation et al. 2004), retrospective longitudinal studies have identified weaknesses on measures of phonological awareness in kindergarten and at school entry, despite intact skills later in development (Catts et al. 2006; Elwér et al. 2015; Nation et al. 2010). This opens the possibility that early phonological weaknesses may increase a child's vulnerability to later problems.

Within a multiple risk framework, it is possible to construe risk both in terms of the number of weaknesses (breadth) and their severity (depth). In contrasting these approaches, Hayiou‐Thomas et al. (2021) counted the number of below‐average scores on preschool assessments of language, speech, nonverbal ability, phonological awareness, and letter knowledge, alongside a family risk indicator for reading/language difficulties. Children with more risk factors were at higher risk of poor reading comprehension outcomes than those with no or few risk factors. Adding information on the severity of impairment on each risk factor did not substantially improve the prediction of long‐term outcomes. These preliminary findings indicate that it is breadth rather than depth of risk that is associated with later reading comprehension.

### 1.3 The Present Registered Report

We examined early predictors of reading CW using data from the Avon Longitudinal Study of Parents and Children (ALSPAC). In a previous study, we used mixture modelling to identify a profile of children with CW at age 9 (James et al. 2023). We found little evidence of the traditionally selected profile with below‐average comprehension in the context of good word reading and decoding, but by controlling for overall reading ability, we extracted and validated a profile who had weak comprehension relative to their level of word reading and decoding. Further consideration of this group enables us to consider children who have unexpectedly poor reading comprehension across the reading spectrum, without imposing arbitrary boundaries. Study 1 embraced this dimensional approach to test whether these children with CW (*n* = 879) had language weaknesses at 15, 24 and 38 months, relative to peers later identified as not having reading weaknesses (*n* = 4130). We then tested the extent to which early language weaknesses predict later comprehension outcomes, and whether early risk is exacerbated by the early language and communication environment in which it occurs. That is, we might predict that a child with poor language in a rich communicative environment will be more likely to catch up prior to school entry (48–59 months) than a child who has poor language in a less interactive and resourceful environment, with implications for later literacy success (Stothard et al. 1998).

Within the ALSPAC cohort, a subsample (*Children in Focus*) completed additional assessments at various time points. Study 2 focused on the children with CW who fell within this subsample (*n* = 125) and considered receptive and expressive language, phonemic awareness, verbal memory, and nonverbal ability from 25 to 61 months of age, relative to controls (*n* = 561). Building on the findings of Hayiou‐Thomas et al. (2021), we asked whether the breadth versus depth of difficulties better predicts later comprehension group membership. Given that the children with CW span a range of reading abilities, we examined whether breadth and depth predict the severity of reading impairment within this group, as implied by concurrent analyses in mid‐childhood (James et al. 2023). Both studies were planned and preregistered in advance of data analysis (i.e., we had only inspected data availability at this stage). The approved Stage 1 protocol is available at https://osf.io/hyzu5.

## 2 Study 1: Early Language

Using preschool measures of language, we asked: (1) for children identified as having CW in mid‐childhood, do they show early language weaknesses relative to peers? and (2) To what extent does preschool language predict later comprehension outcomes, and is prediction improved by the early language and communicative context in which difficulties occur?

### 2.1 Sample

ALSPAC recruited 14,541 pregnant women in the former Avon area (UK) between April 1991 and December 1992, from whom 13,988 offspring were alive at 1 year. A further 913 eligible children were recruited into the study at age 7 years, increasing the total sample size to 14,901. The offspring have been studied ever since via a wide range of questionnaires and clinic assessments (Boyd et al. 2013; Fraser et al. 2013; Northstone et al. 2019). The study website contains details of all the data that is available through a fully searchable data dictionary and variable search tool (http://www.bristol.ac.uk/alspac/researchers/our‐data/). Ethical approval for the study was obtained from the ALSPAC Ethics and Law Committee and the Local Research Ethics Committees. Informed consent for the use of data collected via questionnaires and clinics was obtained from participants following the recommendations of the ALSPAC Ethical and Law Committee at the time.

We used mixture modelling to identify different profiles of readers in ALSPAC, based on measures of reading accuracy, reading comprehension, and listening comprehension (James et al. 2023; Table 1). We included all participants who completed the Neale Analysis of Reading Ability (NARA‐II; Neale 1997) during a clinic visit at age 9 years (*n* = 6935; *n* = 6846 following the removal of twin pairs to address non‐independence). We note that children of this age can be in school years 4 or 5 in the UK education system, but that this information was not available in the dataset to consider potentially different amounts of reading instruction. In the current study, we detail the early language skills of those identified as having CW (*n* = 947; James et al. 2023). We compared their preschool language skills to children not later identified as having a reading weakness (i.e., excluding the profile with word reading weaknesses). We excluded participants who did not have scores on any of the preschool language assessments (e.g., because they were later recruits to the study, or due to low questionnaire response rates in the early years; 8% of the subsample). This left 879 children with CW and 4130 TD readers, as summarised in Table 1.

**TABLE 1:** Sample‐standardised descriptive statistics for the reading accuracy and comprehension measures used in identifying reading profiles at age 8–9 years.

	Children with comprehension weaknesses		Typically developing readers	
	*M*	(*SD*)	*M*	(*SD*)
Reading accuracy Item accuracy^a^	99.63	(15.10)	103.34	(13.15)
NARA^b^ passage accuracy	106.01	(14.19)	100.28	(13.86)
Comprehension NARA^b^ passage comprehension	92.71	(12.39)	102.78	(14.37)
WOLD^c^ listening comprehension	95.57	(13.64)	100.25	(15.12)

*Note*: Scores reflect performance age‐standardised on the sample entered into the latent profile analysis (*n* = 6846).

^a^ Combined real and nonword reading scores (Nunes et al. 2003).

^b^ Neale Analysis of Reading Ability Second Revised British Edition (NARA‐II; Neale, 1997).

^c^ Wechsler Objective Language Dimensions (Rust 1996).

The groups included slightly more females than males (CW: 53.36% female; TD: 51.40% female), reflecting both small sex differences in attendance at the assessment clinics and a higher prevalence of males in the group with word reading weaknesses excluded from the present analyses. The sample was predominantly white, based on maternal questionnaire responses (CW: 91.70% white, 4.21% non‐white, 4.10% not recorded; TD: 91.77% white, 3.80% non‐white; 4.43% not recorded).

These groups reflect the same sample that were recently described in a registered report study of educational and occupational outcomes in adolescence and early adulthood (James et al. 2024). However, we certify that we had not conducted any analyses on the outcome variables included in this report at the time of Stage 1 acceptance. Our research questions were outlined in a preregistration used to select the reading subgroups (https://osf.io/4zahf), but were not specified in detail at this stage.

### 2.2 Measures

#### 2.2.1 Language at 15 Months

Parents completed components of the MacArthur Infant Communication Questionnaire (Fenson et al. 1994) in a postal questionnaire sent at 15 months. The Phrases Understood subscale asks parents to mark yes/no in response to whether their child understands simple phrases (e.g., *come here, don't do that*), with responses summed to a maximum score of 12. In the Vocabulary subscale, parents mark whether their child understands (Score 1), understands and says (Score 2) or neither (0) for 134 words, summed to a maximum score of 268.

#### 2.2.2 Language at 24 Months

Parents completed a selection of items from the MacArthur Toddler Communication Questionnaire (Fenson et al. 1994) in a postal questionnaire sent at 24 months. The Vocabulary subscale included 123 words scored as above, summed to a maximum score of 246. The Grammar subscale included assessment of both regular and irregular plurals and tenses. Regular inflections were rated as not yet (0), sometimes (1), or often (2), summed to a maximum value of 8. Irregular inflections included 25 items were marked as understands (1), says (2) or neither (0), summed to a maximum value of 50.

#### 2.2.3 Language at 38 Months

Parents completed Vocabulary and Grammar items from the MacArthur Toddler Communication Questionnaire, as before. The Grammar component at this age included an additional 12 word‐combination items, rating the grammatical complexity of phrases that the child typically produces.

#### 2.2.4 Early Language and Communication Environment (ELCE)

This has been computed in previous studies using ALSPAC (Gibson et al. 2021; Roulstone et al. 2011). Using items from parent questionnaires completed at 18–24 months, five subscales were created to capture mother‐child direct teaching, mother‐child activities^2^, other‐child interactions, resources, and other activities. These subscales were then used to create a continuous latent variable. While the relation between these variables and preschool language has already been demonstrated in the ALSPAC sample (Law et al. 2019; Roulstone et al. 2011), we have no prior knowledge of its correspondence with reading at 9 years, nor its relation within the current subsamples.

### 2.3 Planned Analyses

In all analyses, we treated the two groups of interest (CW; TD) as fixed categories, based on each individual's most likely group membership from the previous mixture model (James et al. 2023). Although this approach cannot incorporate classification uncertainty (Ferguson et al. 2020), it allowed us to focus on our key comparisons of interest in a way that is consistent with Study 2 (for which three‐step approaches building on the existing mixture model would not have been possible with the reduced sample). This approach was also justified by the large sample and cross‐validation of our previous model, acceptable levels of entropy (0.79), and high classification probabilities in the group of interest.

### 2.4 Q1) Do Children with Comprehension Weaknesses Show Language Weaknesses at 15, 24 and 38 Months?

Given that slightly different language assessments were used at each age, and that there may have been variability in the child's age when their parents completed the questionnaire, we created an age‐normed language composite at each time point. This was created by (1) regressing each subscale on the age at which the questionnaire was completed; (2) standardising the residuals (based on all 6846 participants included in the original mixture model; *n* = 6278 once participants without preschool language data had been excluded); then (3) averaging across the subscales^3^. Note that published test norms could not be used, given that only a subset of items were administered in the study. For the language composite at each age, we tested for differences between children with CW and TD readers using independent samples *t*‐tests, and calculated Cohen's *d* to describe the effect size.

### 2.5 Q2) To What Extent Does Preschool Language Predict Later Comprehension Outcomes, and Is Prediction Improved by the Early Language and Communicative Context in which Difficulties Occur?

Logistic regression was used to test the predictive validity of preschool language assessments for determining group outcomes at age 9 years. Based on previous studies (Justice et al. 2013), we anticipated that the largest group differences in language will be observed at the latest assessment point (38 months). Thus, we used the language composite at 38 months as a predictor of group classification. We report the model parameters and Nagelkerke's Pseudo‐*R* ^2^ to index model fit. To assess the predictive power of the model, we report the in‐sample predictive accuracy, sensitivity, specificity, positive and negative predictive values (PPV, NPV), and area under the curve (AUC).

To address the second hypothesis that prediction improves when including the early language and communicative context of language difficulties, subsequent models added (1) the ELCE index to test the relevance of this variable as a predictor, and (2) its interaction with the language composite to examine multiplicative risk on comprehension outcomes. Model comparisons tested for improvement in model fit and, if apparent, we report the predictive measures as above. A significant interaction would be further probed using a simple slopes analysis, comparing the slope of the language composite at the mean of ELCE with −1 SD and +1 SD.

### 2.6 Missingness

There is no missingness on the grouping variable since inclusion in the former analysis depended on the availability of reading data at age 9. We anticipated some missingness on the preschool language assessments and ELCE subscales, as these measures relied on parental questionnaire returns in early childhood. We therefore inspected the distribution of missingness in relation to comprehension group and alongside measures known to be associated with missingness.

We addressed missingness via multiple imputation, using the *mice* package in R (Buuren and Groothuis‐Oudshoorn 2011). Alongside the analytic variables, the imputation model included auxiliary variables hypothesised to predict either missingness and/or the missing values: sex, ethnicity, verbal and nonverbal IQ at age 8, reading accuracy and comprehension scores at age 9, and several parental/maternal factors (home ownership, marriage, age at birth, occupational class, education, financial difficulties, postnatal depression). We started by setting the number of imputations (*m*) to the highest proportion of missing data in any given variable, and then added further imputations if the loss of precision (Fraction of Missing Information/*m*) was >1% (White et al. 2011; Woods et al. 2021). We also re‐ran the analyses using pairwise deletion; this is reported in full in supplementary materials (Tables S1–S3), and any differences in interpretation are discussed in the main manuscript.

### 2.7 Statistical Power

The sample size was pre‐determined by the availability of data, but power analyses estimated the effect sizes that we are powered to test. For Question 1, we used the *pwr* package (Champely et al. 2017) to determine that we had 90% power to detect a small effect size of *d* = 0.12 at *α* = 0.05. This indicates sufficient power to detect the differences estimated by Justice et al. (2013).

For Question 2, we used a data simulation approach to estimate the power to detect differences in the probability of comprehension group outcomes depending on preschool language and the ELCE. We used 500 simulated datasets, generated using a correlation of 0.3 between the predictor variables (based on correlations reported between related assessments of home literacy and early language, e.g., Weigel et al. 2006). For the first hypothesis testing prediction from language alone, we estimated 90% power to detect a −0.14 change in log odds associated with a 1 SD increase in language ability (Figure S1). This corresponds to a 13.39% decrease in the odds of having CW, indicating sufficient power to detect the effects reported in the literature (e.g., Justice et al. 2013; Hayiou‐Thomas et al. 2021).

For the second hypothesis regarding the role of the ELCE, we fixed the language coefficient to −0.695 (as reported by Hayiou‐Thomas et al. 2021) and examined power across varying ELCE effect sizes. With no interaction, we estimated 90% power to detect a −0.15 change in log odds associated with a 1 SD increase in the ELCE (Figure S2; corresponding to an odds ratio of 0.86). Assuming that the ELCE main effect would be larger than the interaction but smaller than the language coefficient, we estimated power to detect the language‐by‐ELCE interaction across ELCE effect sizes ranging between −0.2 and −0.5. The smallest interaction coefficient with 90% power was 0.15 (Figure S3). In sum, this analysis was powered to detect relatively small effect sizes (Chen et al. 2010). We note that statistically significant effects are not always practically meaningful in large datasets and focus on the effect size and uncertainty around the estimate.

## 3 Study 1 Results

### 3.1 Q1) Do Children with Comprehension Weaknesses Show Language Weaknesses at 15, 24 and 38 Months?

Inspection of the age‐adjusted scores indicated that the group who went on to show weak comprehension in mid‐childhood had slightly lower language scores at each assessment point, relative to children were not later identified as having specific reading weaknesses (Table 2). At 15 months, group differences were minimal and not statistically significant (*t* = −1.18, *p* = 0.235, *d* = 0.04). They had increased by 24 months (*t* = −3.25, *p* = 0.001, *d* = 0.12), and were largest at 38 months (*t* = −4.86, *p* < 0.001, *d* = 0.17). In supplementary analyses with pairwise deletion, differences were only statistically significant at 38 months (Table S1). However, it is notable that the effect size remains small even by at this later assessment.

**TABLE 2:** Availability and summary of early language variables according to the two reading profiles.

	Children with comprehension weaknesses			Typically developing readers		
	% data	*M*	(*SD*)	% data	*M*	(*SD*)
Language composite 15 m	95.52	−0.02	(0.93)	96.70	0.02	(0.89)
Language composite 24 m	84.67	0.02	(0.96)	83.50	0.07	(0.91)
Language composite 38 m	89.49	−0.03	(0.82)	88.40	0.07	(0.74)
ELCE	98.14	−0.02	(0.63)	98.41	0.02	(0.60)

### 3.2 Q2) To What Extent Does Preschool Language Predict Later Comprehension Outcomes, and Is Prediction Improved by the Early Language and Communicative Context in which Difficulties Occur?

While the logistic regression model indicated that language at 36 months was a significant predictor of comprehension group outcomes (*OR* = 0.82, *p* < 0.001), model fit was poor (*R* ^2^ = 0.007). Table 3 presents model classification performance at two thresholds: the optimal threshold determined by the ROC curve (balancing sensitivity and specificity), and the threshold corresponding 90% sensitivity^4^. The latter was chosen on the basis of previous work in predicting reading disorders (Puolakanaho et al. 2007; Thompson et al. 2015), and aligns with the desired sensitivity for clinical screening tools. However, in‐sample accuracy was poor at both thresholds: correctly predicting outcomes for 65% of the sample at the optimal threshold, and only 26% at 90% sensitivity. ROC analyses supported the conclusion that model prediction was poor regardless of the threshold selected (AUC = 0.54, 95% CI [0.52, 0.57]).

**TABLE 3:** Classification performance when using early language ability to predict comprehension group outcomes.

Threshold justification	Probability threshold	Predictive accuracy	Sensitivity	Specificity	Positive predictive value	Negative predictive value
ROC optimal	0.18	0.65	0.38	0.70	0.21	0.84
90% sensitivity	0.16	0.26	0.90	0.12	0.18	0.85

In the second model, we added the ELCE index alongside its interaction with early language. Model comparison indicated no improvement in model fit (*p* = 0.944), reflecting no main effect (*p* = 0.760) or interaction (*p* = 0.839) of the additional predictors.

## 4 Study 1 Summary

In Study 1, we examined when early language weaknesses can be observed in children who go on to have relatively weak comprehension skills at age 9. While group differences were apparent at 24 and 38 months, they remained small and unable to predict subsequent comprehension on an individual level—even when combined with environmental measures, as captured by the ELCE index.

## 5 Study 2: Breadth Versus Depth of Early Weaknesses

Study 2 looked beyond language to examine whether co‐occurring cognitive weaknesses increase the likelihood of later CW. Using data from a subsample who completed more in‐depth assessments, we asked: (1) whether the number of early risk factors predicts later comprehension group status; (2) whether additional information on the severity of early risk factors enhances predictive accuracy; and (3) whether the number of early risk factors predicts the severity of later reading difficulties.

### 5.1 Sample

Study 2 focused on a subset of the Study 1 sample who took part in additional Children in Focus (CiF) clinic assessments between 4 and 61 months of age. The CiF sample reflects 10% of the cohort (*n* = 1432), who were invited at random from the final 6 months of ALSPAC births in 1992 (excluding those who had moved out of the area or were taking part in another study). From the two groups described above, 126 children with CW and 571 TD readers were part of the CiF sample. After excluding participants without any relevant assessments, the final sample comprised 125 children with CW and 561^5^ TD readers for analysis.

### 5.2 Measures

These hypothesised domains formed our starting point, but note that the factor structure was altered during the first stage of analysis according to model fit (described in detail below). We selected these measures as multiple indicators of relevant constructs. While the measures span multiple age assessments, we combined across these as multiple indicators of early ability.

#### 5.2.1 Receptive Language

This was captured in early childhood using the MacArthur CDI Vocabulary subscale at 38 months (scored for ‘Understands’ only)^6^, as well as the Verbal Comprehension subscale of the Reynell Developmental Language Scale (Reynell 1977) at 25 and 61 months. In the latter, children are asked to interact with small toys according to increasingly complex instructions (e.g., *where is the horse? Put the white button underneath the cup*).

#### 5.2.2 Expressive Language

This was captured by the MacArthur CDI Vocabulary subscale (scored for ‘Says’ only) and Word Combination score at 38 months, alongside two clinic assessments. At 49 months, children completed the vocabulary subtest of the Wechsler Pre‐school and Primary Scale of Intelligence—Revised (UK) edition (WPPSI; Wechsler 1990), which includes picture naming and verbal definition items. At 61 months, verbal expression was measured using the Bus Story Test (Renfrew 1997). Children listen to a story accompanied by pictures, and are asked to retell the story using the pictures as support. Responses are scored for information content and sentence length.

#### 5.2.3 Phonemic Awareness

At 61 months, children were presented with 10 sets of three‐line drawings and were asked to identify which two began with the same consonants (Byrne and Fielding‐Barnsley 1993). Scores were summed.

#### 5.2.4 Verbal Short‐Term Memory

This was assessed at 49 months and 61 months using the Digit Span Test (Gathercole and Pickering 2000) in which children hear spoken digit sequences of increasing lengths, and repeat them back. The total score reflects the number of sequences correctly recalled. At 61 months children completed a nonword repetition task. They were asked to repeat back nonwords of 2–5 syllables, placing demands on phonological processing and verbal short‐term memory. Scores were summed over the 40 items.

#### 5.2.5 Nonverbal IQ

This was measured at 49 months using five subtests of the WPPSI: object assembly, geometric design, block design, mazes and picture completion.

#### 5.2.6 Early Language and Communication Environment (ELCE)

The ELCE was measured as described in Study 1.

### 5.3 Planned Analyses

#### 5.3.1 Factor Structure

The proposed factor structure comprised five latent variables (receptive language, expressive language, verbal memory, nonverbal IQ, ELCE) and a single observed score for phonemic awareness. However, as we had no prior experience with these variables in ALSPAC, we considered whether model fit might be poor. For example, receptive and expressive language may reflect a single language factor, and nonword repetition may also load with language and phonemic awareness. Thus, we tested alternative factor structures, and considered theoretically appropriate adjustments guided by modification indices to optimise model fit. Acceptable model fit was determined by at least three of the four following criteria being met: CFI > 0.90; TLI > 0.90; RMSEA < 0.08; and SRMR < 0.08. Any further changes once this criterion has been met would have required strong theoretical justification. All tested models are presented in an annotated output file on the Open Science Framework (https://osf.io/f4kjv).

We planned that, should model fit remain poor at this stage, we would inspect each of the observed measures for distribution and range and consider transformation or removal of variables where appropriate to enable a successful fit. If it was not possible to reconcile the measures within an appropriate theoretical framework, then there was scope for proceeding with the analyses using a smaller number of observed measures; however, this would be reported as an exploratory analysis and the limitations made transparent in the discussion.

After determining an appropriate way of structuring the variables for analysis, we extracted factor scores for each participant using the ten Berge method (Logan et al. 2022; ten Berge et al. 1999), as implemented in the *SEMinR* package (Hair et al. 2021). These formed the ability scores referred to in the analysis plans described next. The use of factor scores (versus individual indicators) aids in addressing the high correlation between certain measures, and allowed us to interpret the distribution of risk factors across different domains of ability.

#### 5.3.2 Q1) Does the Number of Early Risk Factors Predict Later Comprehension Group Status?

To consider whether a breadth of risk factors predicts later comprehension group status, each ability score was dichotomised into risk present (1) or absent (0), using a relatively liberal threshold of the 25th percentile. We describe the number of risk factors present for each group, and their distribution across the domains assessed. A Spearman's correlation between the number of risk factors and comprehension group outcome tested the hypothesis that the breadth of risk factors predicts later comprehension impairment. To compare our results to those reported by Hayiou‐Thomas et al. (2021), we then computed odds ratios to capture the extent to which few (1–2) or many (3+) risk factors increase the likelihood of a poor comprehension outcome relative to no risk factors.

#### 5.3.3 Q2) Does Depth Versus Breadth of Impairments Enhance Predictive Accuracy?

To test whether the breadth of impairments is as good a predictor of later comprehension outcomes as their severity, we compared the fit of logistic regression models using categorical risk versus continuous ability predictors. Model 1 used the number of risk factors as a predictor of later comprehension group status. Model 2 included the continuous ability scores as predictors in the logistic regression model. As in Study 1, we report the model parameters and Nagelkerke's Pseudo‐*R* ^2^ to index model fit, alongside in‐sample predictive accuracy, sensitivity, specificity, positive and negative predictive values, and AUC. The superiority of either model over the other can be inferred from non‐overlapping 95% confidence intervals for the AUC estimate.

#### 5.3.4 Q3) Does the Number of Early Risk Factors Predict the Severity of Later Comprehension Outcomes?

This final analysis focused on the children identified as having CW (*n* = 125). This sample has weak reading comprehension relative to their decoding skills, but spans a range of reading ability. We tested the hypothesis that the breadth of risk factors predicts the severity of reading impairment along this continuous scale. First, we correlated the number of risk factors present with the standardised reading comprehension scores from the NARA‐II at age 9. A negative correlation would support the hypothesis that the breadth of early risk factors influences the severity of later comprehension impairment. Second, we compared the fit of linear regression models using categorical risk versus continuous ability predictors (as per Q2 above), but with reading comprehension score as the outcome variable. We report adjusted *R* ^2^ for each, and infer the better‐fitting (i.e., most efficient) model by comparing the corrected AIC values and Akaike weight.

### 5.4 Missingness

Given that the predictor variables are based on factor scores, missingness was dealt with primarily by using full information maximum likelihood estimation in the CFA. This approach is favoured over multiple imputation for simplicity, given the lack of advised methodology for pooling factor score estimates. Any values missing after factor score creation (e.g., for single‐item measures) could have been addressed via multiple imputation, following the same principles as Study 1, however, this was not necessary. We used the same auxiliary variables to assess the missing at random assumption, with the addition of nonword repetition and digit span variables from the age 8 clinic given the additional verbal memory measures in this analysis. However, to reduce computational complexity, only variables that predicted missingness or missing values beyond the variables already included in the model could be included at the CFA stage.

### 5.5 Statistical Power

For Question 1, we estimated 90% power to detect a correlation of *r* = 0.12 between the number of risk factors and group outcomes, indicating sufficient power to detect the relationship found by Hayiou‐Thomas et al. (*r* = 0.40). Based on their finding that 4.94% of children with no early risk factors went on to have comprehension difficulties and the proportions of the sample with few/many risk factors (35%, 24%), we estimated 90% power to detect a 0.09 increase in the probability of comprehension difficulties associated with the presence of risk factors (Figure S4).

For Question 2, we considered the power to detect a difference in the AUC values from Model 1 (number of risk factors) and Model 2 (continuous predictors). We simulated a dataset based on the parameters reported by Hayiou‐Thomas et al. and estimated the binormal parameters in order to conduct a two paired ROC curve power calculation (using package *pROC*; Robin et al. 2011). With our pre‐determined sample size, we estimated 90% power to detect an AUC difference of 0.08 (Figure S5).

For Question 3, we computed that we have 90% statistical power to detect an association of *r* = 0.28 between the number of risk factors and severity of comprehension difficulty within the group with CW. This effect size is similar in magnitude to correlations reported between early language and later reading comprehension skill measured continuously (e.g., Lee 2011), providing a good indication of whether breadth can outperform the single best indicator currently known. The two linear regression models are powered to detect small‐medium effect sizes: Model 1 (number of risk factors) *f* ^2^ *=* 0.10; Model 2 (based on six continuous predictors) *f* ^2^ = 0.15.

## 6 Study 2 Results

### 6.1 Factor Structure

Inspection of the data indicated that missingness could be considered random, dependent on the planned variables, with no further auxiliary variables included. The 6‐factor model (expressive language, receptive language, phonemic awareness, verbal short‐term memory, nonverbal IQ, ELCE) was not supported by the data, but issues were resolved by collapsing the expressive and receptive language variables into a single language factor. Guided by modification indices, we added two covariances to improve model fit: between the two Bus Story scores (information content, sentence length), and between the ‘says’ and ‘understands’ scores of the MacArthur CDI vocabulary assessment. This model met our criteria for model fit (CFI = 0.92, TLI = 0.90, RMSEA = 0.04, SRMR = 0.05), and we proceeded to extract factor scores for analysis. The full model is presented in Table S4.

### 6.2 Q1) Does the Number of Early Risk Factors Predict Later Comprehension Group Status?

**FIGURE 1:**
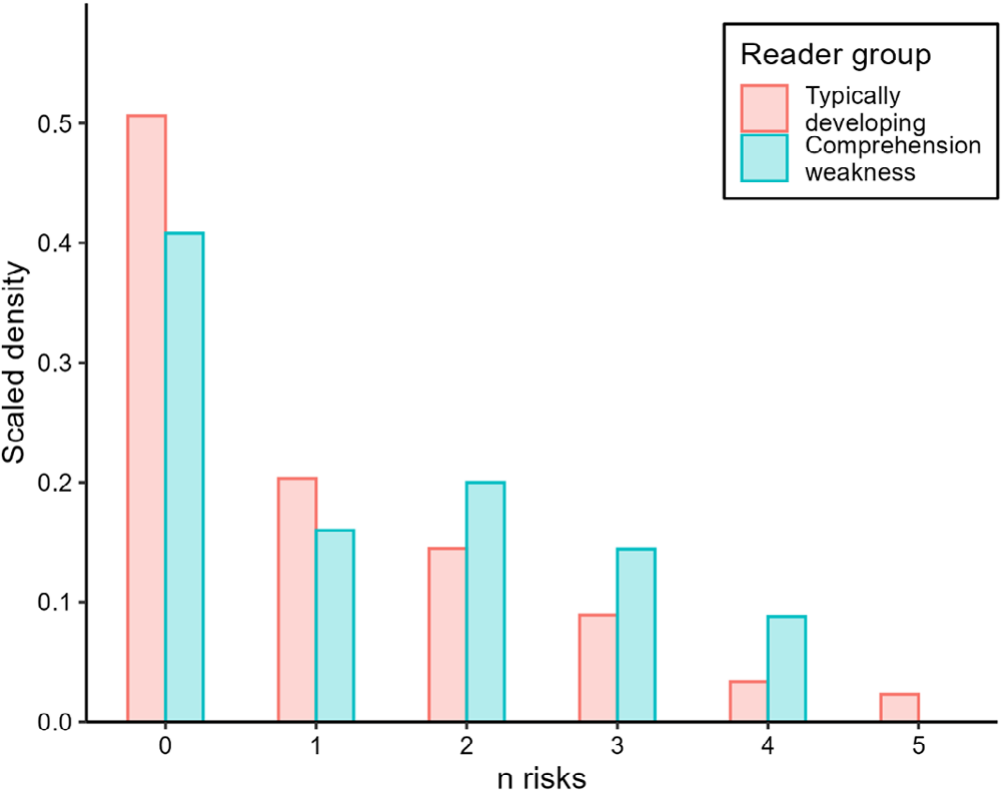
Cumulative risk index for children with and without comprehension weaknesses.

Each child was considered as having a ‘risk factor’ if their ability score was below the 25th percentile. Children with CW showed a higher accumulation of risk factors compared to children without reading weaknesses (Figure 1): a higher proportion of TD readers had 0–1 risks in early childhood than children with CW, whereas a higher proportion of children with CW had 2–4 risks. These risks were distributed across domains, such that a higher proportion of children with CW experienced each risk than those without reading difficulties. (Table 4). The biggest group differences were observed in language (32.8% vs. 21.75%), closely followed by phonemic awareness (28% vs. 19.07%). Correlations between risk factors ranged from 0.09 to 0.45 (Table 5).

**TABLE 4:** Distribution of risk factors and average scores across domains for children with and without comprehension weaknesses.

	Language		Verbal STM		Nonverbal IQ		ELCE		Phonemic awareness	
Proportion with risk factor (%)										
Typically developing readers	21.75		22.46		23.89		13.90		19.07	
Comprehension weaknesses	32.80		27.20		29.60		16.80		28.00	
Mean (SD) severity										
Typically developing readers	0.07	(1.01)	0.05	(1)	0.04	(0.99)	−0.01	(1.01)	0.06	(0.97)
Comprehension weaknesses	−0.23	(0.98)	−0.18	(1.04)	−0.12	(1.05)	−0.07	(1.04)	−0.08	(0.98)

Abbreviations: ELCE, Early Language and Communication Environment; STM, short‐term memory.

**TABLE 5:** Phi correlations between risk factors.

	Language	Verbal STM	Nonverbal IQ	ELCE	Phonemic awareness
Language	1	0.30	0.45	0.23	0.35
Verbal STM		1	0.22	0.09	0.32
Nonverbal IQ			1	0.20	0.30
ELCE				1	0.17
Phonemic awareness					1

Abbreviations: ELCE, Early Language and Communication Environment; STM, short‐term memory.

The Spearman's correlation indicated that the number of early risk factors was weakly correlated with later comprehension group (*r*(684) = 0.10, *p* = 0.007). Having 1–2 risk factors did not significantly increase the odds of having later CW, OR = 1.29, 95% CI [0.83, 2.00] (Table 6). Having three or more risk factors, however, nearly doubled the odds, OR = 1.97, 95% CI [1.16, 3.29]. Only 15% of children with no risk factors went on to show CW in mid‐childhood, compared to 26% of those with three or more.

**TABLE 6:** Breadth and depth models in predicting reading comprehension weaknesses in mid‐childhood.

	*β*	SE	*Z*	*p*	OR	95% CI
Breadth model						
(Intercept)	−1.72	0.15	−11.29	<0.001	0.18	[0.13, 0.24]
1–2 risks	0.25	0.22	1.12	0.264	1.29	[0.83, 2.00]
3+ risks	0.68	0.26	2.57	0.010	1.97	[1.16, 3.29]
Depth model						
(Intercept)	−1.53	0.10	−15.06	<0.001	0.22	[0.18, 0.26]
Language	−0.33	0.16	−2.05	0.041	0.72	[0.53, 0.99]
Verbal STM	−0.10	0.12	−0.81	0.416	0.90	[0.71, 1.15]
Nonverbal IQ	0.07	0.13	0.52	0.601	1.07	[0.82, 1.40]
ELCE	0.07	0.11	0.61	0.540	1.07	[0.87, 1.33]
Phonology	0.02	0.12	0.15	0.881	1.02	[0.80, 1.29]

Abbreviations: ELCE, Early Language and Communication Environment; STM, short‐term memory.

### 6.3 Q2) Does Depth Versus Breadth of Impairments Enhance Predictive Accuracy?

While the model based on the breadth of risk factors showed that having three or more early risks was associated with CW in mid‐childhood, the overall model fit was poor (Nagelkerke's Pseudo‐*R* ^2^ = 0.02). As with Study 1, we did not preregister decision thresholds, but examined classification performance at the optimal threshold according to the ROC curve. The model correctly predicted 52% of cases at this point (Table 7). It was not possible to assess performance at the 90% sensitivity threshold due to the categorical nature of the predictors.

**TABLE 7:** Classification performance in using breadth versus depth of risk factors to predict comprehension group outcomes.

Model and threshold justification	Probability threshold	Predictive accuracy	Sensitivity	Specificity	Positive predictive value	Negative predictive value
Breadth						
ROC optimal	−1.59	0.52	0.59	0.51	0.21	0.85
Depth						
ROC optimal	−1.27	0.73	0.31	0.82	0.28	0.84
90% sensitivity	−1.88	0.27	0.90	0.13	0.19	0.85

*Note*: It was not possible to select a threshold at 90% sensitivity in the breadth model given then constrained categorical nature of the predictors. The ROC optimal threshold was the closest sensitivity value that was not 1.

The second model used continuous ability scores to test whether the severity of impairments was a better predictor of comprehension outcomes. The risk index calculated in this model was correlated *r(*684) = 0.62 with the number of risk factors, indicating potential for differential prediction. As summarised in Table 6, inspection of the model parameters indicated that only language was associated with reading outcomes (OR = 0.72, 95% CI [0.53, 0.99]). Model fit remained poor (Pseudo‐*R* ^2^ = 0.02). Classification statistics indicated slightly higher accuracy at the ROC optimal threshold (73%) than was observed for the breadth model, but this suffered poor sensitivity (0.31). Correspondingly, accuracy at the 90% sensitivity threshold was poor at 27% (Table 7). In comparing the two models, the AUC statistics showed similarly poor classification for both the breadth (0.56, 95% CI [0.51–0.62]) and depth (0.58, 95% CI [0.52–0.64]) of risk factors.

### 6.4 Q3) Does the Number of Early Risk Factors Predict the Severity of Later Comprehension Outcomes?

Within the group with reading CW, the number of risk factors was significantly correlated with their reading comprehension scores (*r*(123) = −0.37, *p* < 0.001). That is, the breadth of early risk factors predicts the severity of reading comprehension impairments. The breadth model again showed that having three or more risk factors was most closely associated with poor reading outcomes, corresponding to an average decrease of 10.22 standard score points in reading comprehension, 95% CI [−14.51, 5.93] (Table 8). The adjusted *R* ^2^ indicated that the breadth of risk factors could account for 14.07% variation in comprehension impairments.

**TABLE 8:** Breadth and depth models in predicting severity of reading comprehension impairment in mid‐childhood.

	*β*	SE	95% CIs	*Z*	*p*
Breadth model					
(Intercept)	98.08	1.30	[95.50, 100.66]	75.18	<0.001
1–2 risks	−3.23	1.91	[−7.01, 0.54]	−1.70	0.092
3+ risks	−10.22	2.17	[−14.51, −5.93]	−4.72	<0.001
Depth model					
(Intercept)	96.56	0.77	[95.03, 98.09]	125.32	<0.001
Language	−0.85	1.08	[−2.99, 1.29]	−0.78	0.435
Verbal STM	2.59	0.97	[0.67, 4.51]	2.68	0.009
Nonverbal IQ	1.77	0.85	[0.07, 3.46]	2.07	0.041
ELCE	0.60	0.73	[−0.85, 2.05]	0.82	0.415
Phonology	4.46	0.75	[2.98, 5.94]	5.98	<0.001

Abbreviations: ELCE, Early Language and Communication Environment; STM, short‐term memory.

The severity model with continuous predictors accounted for a higher proportion of variation, 43.98%. This was clearly the better fitting model, with corrected AIC 732.84 versus 917.98 (∆AIC = 185.14). While language ability had been the most robust predictor of the *presence* of relatively weak comprehension in the analyses for Q2, it had no further role to play in predicting the *severity* of comprehension impairments (Table 8). Rather, and in order of effect size, phonemic awareness, verbal short‐term memory, and nonverbal IQ were all associated with the severity of comprehension impairments.

## 7 Study 2 Summary

Children with relatively weak comprehension in mid‐childhood were more likely to have experienced a range of preschool difficulties spanning language ability and context, phonemic awareness, verbal short‐term memory, and nonverbal IQ. Risks in three or more of these domains nearly doubled the likelihood of children experiencing later comprehension problems. However, neither the cumulative breadth of difficulties nor specific ability scores were able to reliably predict which preschool children would go on to be classified in the CW group. Instead, early predictors were better able to capture the variation in reading comprehension skills *within* this group. Our analyses indicate that while early language ability is the most consistent and robust predictor of later comprehension difficulties, it is the additional risks in other domains that increase the likelihood of poor outcomes and impact the severity of impairment.

## 8 General Discussion

It is well‐accepted that some children find reading comprehension particularly difficult, and that this profile of poor reading is associated with language weaknesses more broadly. There is some evidence that early language difficulties might be causally implicated in later difficulties with reading comprehension (Catts et al. 2006; Elwér et al. 2015; Nation et al. 2010), and this makes sense given the relationship between language skills and learning to read more generally (Snowling and Hulme 2021). Equally, however, reading comprehension is a complex trait that draws on multiple factors, and the speculation that there might be different routes to becoming a ‘poor comprehender’ has been offered for some time (e.g., Nation 2005). While this sits comfortably within a multiple risk framework, direct evidence is lacking. Previous longitudinal studies are limited in size and scope, both in terms of the number of children that go on to have later difficulties with reading comprehension, and the range of abilities tested within and beyond language.

In earlier work, we used a data‐driven approach to identify a large sample of 9‐year‐old children with relative CW (*n* = 947; James et al. 2023). This profile was associated with lower levels of oral language and nonverbal ability, and a small subgroup showed more severe comprehension difficulties in the context of additional cognitive impairments. These findings are consistent with poor comprehension (relative to decoding) in mid‐childhood being characterised along a continuum, and with there being multiple risks that influence severity. This registered report describes secondary analyses of the same dataset to address questions about the breadth and depth of risk factors earlier in development, and to investigate longitudinal predictors on comprehension outcomes in mid‐childhood.

Consistent with the concurrent language weaknesses noted above, children with comprehension difficulties in mid‐childhood had lower levels or oral language at 24 and 38 (but not 15) months of age, with group differences increasing in size with age. However, preschool language was a poor predictor of later group membership, with only 65% of children being classified correctly. These findings echo small‐scale findings reported by Justice et al. (2013), both in terms of there being group differences in language in the preschool years, and these being poor predictors of individual outcomes. More generally, these findings are consistent with studies assessing reading outcomes in late talkers (e.g., Duff, Nation et al. 2015). We had predicted that adding an index of the language and communication environment into the model would improve prediction, but this was not the case. Given our sample size, this lack of prediction is unlikely to be a simple consequence of a lack of statistical power. Instead, it is consistent with the idea that factors beyond language are also at play and that additional risk factors need to be considered if prediction is to improve.

Study 2 provided an opportunity to test this idea. Children with comprehension weakness had more risk factors in the preschool years than those without CW. Once again, language was associated with the largest group differences, but elevated levels of risk were seen across all domains, including nonverbal ability, verbal short‐term memory, and phonemic awareness, as well as the language and communication environment. Notably, having three or more risk factors in the preschool years nearly doubled the odds of being categorised into the weak comprehension group in mid‐childhood. Overall, however, sensitivity and specificity levels were low, indicating that it was not possible to accurately identify categorical outcomes for individuals from the number or severity of early risk factors.

There was some evidence that children with more early risk factors had the most severe comprehension impairments later in development, and that the depth of the risk factors (i.e., their severity) was associated with the severity of later comprehension difficulties. Language as a risk factor showed an interesting pattern: it was the strongest predictor of the presence of CW in mid‐childhood, but beyond this, it did not predict the severity of the comprehension impairment. In contrast, while other risk factors were less robust than early language in predicting group membership, phonemic awareness, verbal short‐term memory and nonverbal ability were all associated with the severity of outcomes. Taken together, these findings indicate that the presence of multiple risks is associated with both the presence of later CW and their severity. While lower levels of language put children at risk for later CW, abilities in other cognitive domains may serve as risk or resilience factors in determining the severity of outcomes.

### 8.1 Cognitive Risks for Comprehension Weaknesses

The three risk factors that emerged beyond language are not new in that all have been considered in the experimental literature that has compared groups of ‘poor comprehenders’ with a control group. For nonverbal IQ and verbal working memory, group differences tend to be small and/or variable across studies. This might reflect inadequate sample sizes alongside variation in age, selection criteria, and the measures used across studies. We captured verbal short‐term memory at 4‐ and 5‐years using digit span and nonword repetition. Previous work shows that poor comprehenders in mid‐childhood typically perform well on these tasks (e.g., Nation et al. 2004; Stothard and Hulme 1992). However, when verbal working memory is taxed using more complex measures such as listening span, group differences are and a minority of poor comprehenders show broader working memory difficulties (e.g., Carretti et al. 2009; Pimperton and Nation 2014). Turning to nonverbal ability, when measured or part of a study's selection criteria, poor comprehenders as a group typically show nonverbal IQ in the normal range but lower than the comparison group (Catts et al. 2006; Elwér et al. 2015; Nation et al. 2010) and when individual variation within a selected sample has been explored, some poor comprehenders show deficits in nonverbal IQ (Nation et al. 2002). Thus, there is evidence from previous work consistent with both verbal memory and nonverbal ability being associated with poor reading comprehension. These weaknesses occur in the context of more substantial language difficulties, and note too that language differences maintain even when nonverbal IQ is controlled (Catts et al. 2006; Elwér et al. 2015). Together, these observations sit comfortably with both verbal short‐term memory and nonverbal ability serving as additional risk factors in our study. They also fit with patterns seen in longitudinal studies of developmental language disorder, where higher levels of nonverbal IQ have been described as a protective factor that supports more positive language and literacy outcomes (e.g., Snowling et al. 2000; Stothard et al. 1998).

Difficulties with phonological awareness are not normally associated with the poor comprehender profile in mid‐childhood. Interestingly, however, and in line with phonological awareness at 5‐years being identified as an additional risk fact in our study, three retrospective longitudinal studies all found lower levels of phonological awareness in preschool or at school entry in children who went on to have poor reading comprehension (Catts et al. 2006; Elwér et al. 2015; Nation et al. 2010). In all three, this resolved over time, perhaps as a consequence of learning to read; it might also be that preschool phonological abilities as measured by metalinguistic tasks depend on more general language resources, leading to difficulties for those children with poor language (Metsala and Walley 1998; Torppa et al. 2007). The way children are selected is also likely to influence whether weaknesses in phonological awareness are seen. While all children identified as having comprehension weakness in our study showed weak comprehension relative to word reading, the absolute level of word reading ability varied within the group (cf. poor comprehenders selected in experimental studies based on age‐appropriate word reading). Thus, our observation that variation in phonological skills at school entry was associated with comprehension outcomes might reflect greater variation in word reading ability in our sample. Afterall, children need to be able to read words to comprehend text, and poor phonological ability might therefore carry additional risk via difficulties with word reading itself.

### 8.2 Predicting Poor Outcomes

Although we identified risk factors that were associated with comprehension outcomes and their severity, individual classification remained poor. In fact, classification ability was poorer than in recent work on early prediction of comprehension difficulties: the best AUC achieved in any of our models was 0.58, falling far below the 0.83 reported by Hayiou‐Thomas et al. (2021) and comparable levels in other studies of reading difficulties (e.g., Thompson et al. 2015). However, it is important to note that our study examined a different population of at‐risk children: those who were identified based on *relative* CW across the spectrum of decoding ability, rather than their *absolute* levels of reading performance. While this approach is less typical of research studies, it aligns with the view that risk for reading difficulties is continuous in nature and arguably, it better reflects the varied patterns of strengths and difficulties that teachers are supporting in the classroom. Further, this method of profiling captured more variance in reading performance than profiling on overall ability in earlier work (James et al. 2023) and is significantly associated with education and employment outcomes (James et al. 2024). Clearly, though, variation presents a notable challenge when identifying early markers aligned with a more dimensional view of comprehension problems.

Methodologically, our findings highlight a clear distinction between measures of population‐level associations with outcomes versus prediction of outcomes on an individual basis (Puolakanaho et al. 2007). While the latter reflects a much more stringent test of early risk factors, it is less frequently incorporated in studies examining longitudinal associations in developmental disorders. Our findings show that significant differences observed at a group level may be far from adequate at predicting differences for individuals.

### 8.3 Limitations and Conclusions

Finally, it is worth reflecting on how our conclusions may be affected by the constraints of the data available in ALSPAC. While a good range of language measures were incorporated in the early years—particularly for those children in the CiF subsample for Study 2—other measures were much sparser in nature. A notable omission reflects a lack of executive function measures in the early years. We were able to include two measures of verbal short‐term memory: digit span and nonword repetition. These measures cannot isolate more global weaknesses in nonverbal aspects of memory or in executive functions (e.g., sustained attention, inhibition), which have been previously identified in children with language disorder (e.g., Snowling et al. 2019). Under our more general conclusion that factors beyond language are important to reading outcomes, the contributions of these early cognitive abilities clearly warrant further investigation.

In sum, this registered report capitalised upon secondary data from a birth cohort study to examine early risk factors of later CW at scale. Study 1 successfully replicated the finding that children with CW in mid‐childhood had weaker preschool language ability than their peers, but the language and communication environment did not further enhance associations with outcomes. Study 2 found that the breadth of weaknesses across different cognitive domains may exacerbate risk, and that it is the cognitive factors beyond language that may serve to exacerbate or protect against the severity of outcomes. These findings align with a multiple risk framework, and highlight the value of secondary datasets to capture this breadth often missed by isolated and/or small‐scale studies of reading difficulties.

## Ethics Statement

Ethical approval was obtained from the ALSPAC Ethics and Law Committee and the Local Research Ethics Committees. Informed consent for the use of data collected via questionnaires and clinics was obtained from participants following the recommendations of the ALSPAC Ethical and Law Committee at the time.

## Conflicts of Interest

The authors declare no conflicts of interest.

## Supporting information




**Supporting File 1**: desc70048‐sup‐0001‐SuppMat

## Data Availability

The informed consent obtained from ALSPAC participants does not allow the data to be made freely available through any third‐party maintained public repository. However, data used for this submission can be made available on request to the ALSPAC Executive. The study website contains details of all the data that is available through a fully searchable data dictionary and variable search tool (http://www.bristol.ac.uk/alspac/researchers/our‐data/). To facilitate transparency, annotated output files are available on the OSF (https://osf.io/f4kjv/).

## Endnotes

1 While our questions are motivated by the research on ‘poor comprehenders’, we use the more person‐oriented term of ‘children with comprehension weaknesses’ throughout. This term also better distinguishes our sample of children with comprehension that is relatively weak across the spectrum of decoding ability, as opposed to a distinct category traditionally defined by score thresholds.

2 Gibson et al. (2021) included an ‘Other activities (please specify)’ item in this subscale, which appears to differ from Roulstone et al. (2011). Given that the whole‐sample documentation reports high levels of missingness for this item (85.2%), we chose not to include this item.

3 Although Justice et al. (2013) analysed measures of production and comprehension separately, this option was not available as word production/comprehension was not consistently scored in the ALSPAC dataset.

4 Due to an oversight, we did not specify the threshold to be used at Stage 1. However, the ROC analyses were preregistered at this stage and are not influenced by decision thresholds.

5 At Stage 1, we reported data availability for 562 typically developing readers. This discrepancy was due to two minor coding errors at Stage 1 that were: (1) excluding participants as missing CIF data when it was present, and (2) including participants who had parent‐report language data across any wave, when only the 38‐month time point is relevant for Study 2.

6 We use only the 38‐month CDI for Study 2 as it is the only time point that had robust scoring for ‘Understand’ and ‘Says’ separately. We also anticipate that this oldest time point will have the strongest relationship with later literacy skills.
