# Supplementary material for: Language and Beyond: A Registered Report Examining Single and Multiple Risk Models of Later Reading Comprehension Weaknesses
Source: Dev Sci. 2025 Jul 14;28(5):e70048. doi: 10.1111/desc.70048 (PMC12260278; doi:10.1111/desc.70048)
Supplement: Supplementary file 1 — Supporting File 1: desc70048‐sup‐0001‐SuppMat [file DESC-28-e70048-s001.docx]

**Supporting information**

Language and beyond: a registered report examining single and multiple risk models of later reading comprehension weaknesses

[Figure S1. Statistical power for predicting the probability of group outcomes based on a continuous predictor 2](#_Toc202865359)

[Figure S2. Statistical power for predicting the probability of group outcomes based on two continuous predictors 3](#_Toc202865360)

[Figure S3. Statistical power for predicting the probability of group outcomes based on an interaction between two continuous predictors 4](#_Toc202865361)

[Figure S4. Statistical power for predicting the probability of group outcomes based on a dichotomous risk predictor 5](#_Toc202865362)

[Figure S5. Statistical power for detecting the difference in AUC between two ROC curves 6](#_Toc202865363)

[Table S1. Complete case analysis for differences in language scores at 15, 24, and 36 months 7](#_Toc202865364)

[Table S2. Complete case analysis for predicting reading outcomes on the basis of preschool language and environmental measures 8](#_Toc202865365)

[Table S3. Classification performance when using early language ability to predict comprehension group outcomes (complete case analysis) 9](#_Toc202865366)

[Table S4. Final CFA for Study 2 10](#_Toc202865367)

### Figure S1. Statistical power for predicting the probability of group outcomes based on a continuous predictor


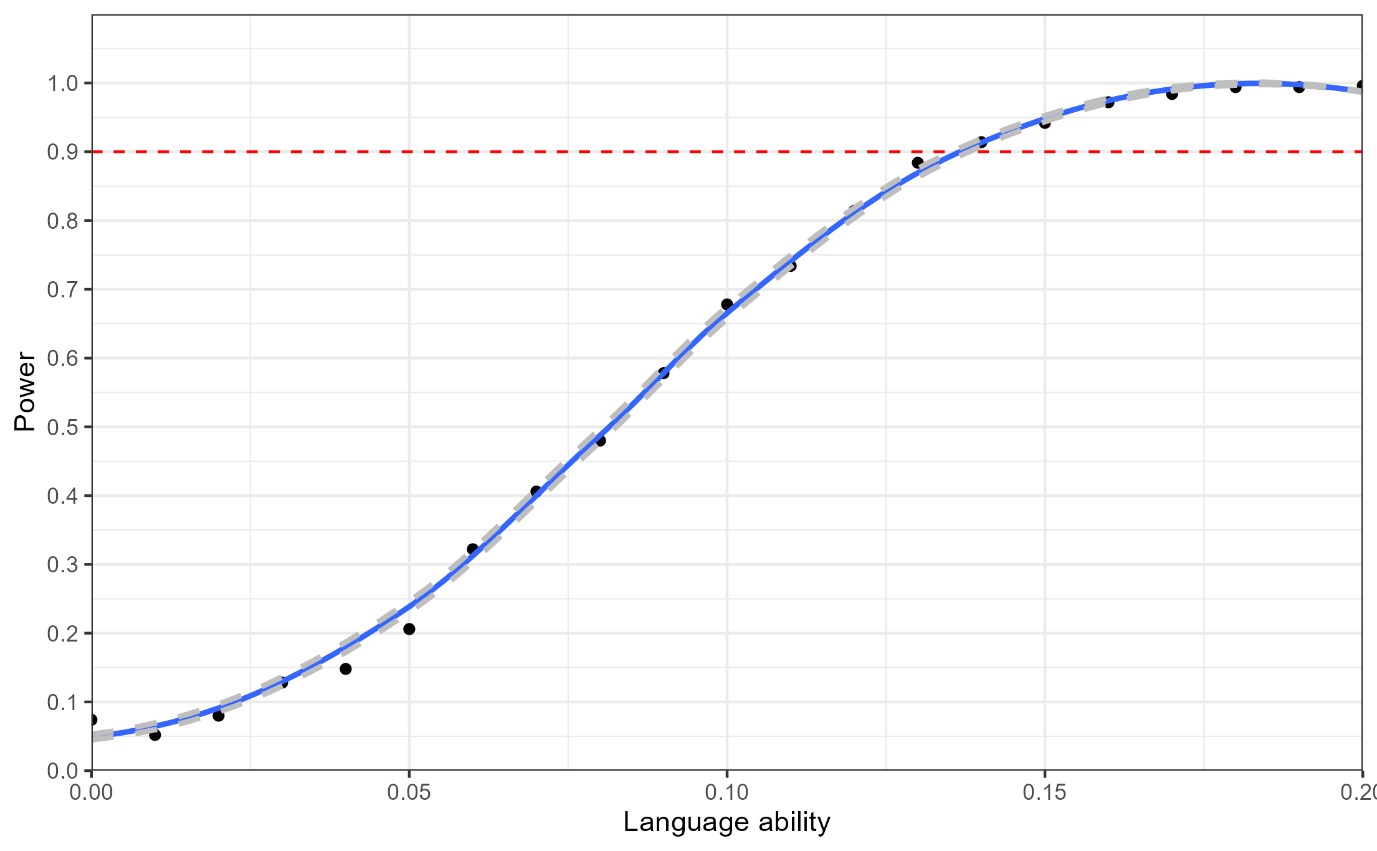


*Note.* Statistical power in logistic regression for *n* = 5009 (proportion of children with comprehension weaknesses = 0.18), with one centered continuous predictor (language ability). Grey dashed lines mark 95% confidence intervals. Power is estimated from 500 simulated datasets; the red dashed line marks the desired power threshold.

### Figure S2. Statistical power for predicting the probability of group outcomes based on two continuous predictors


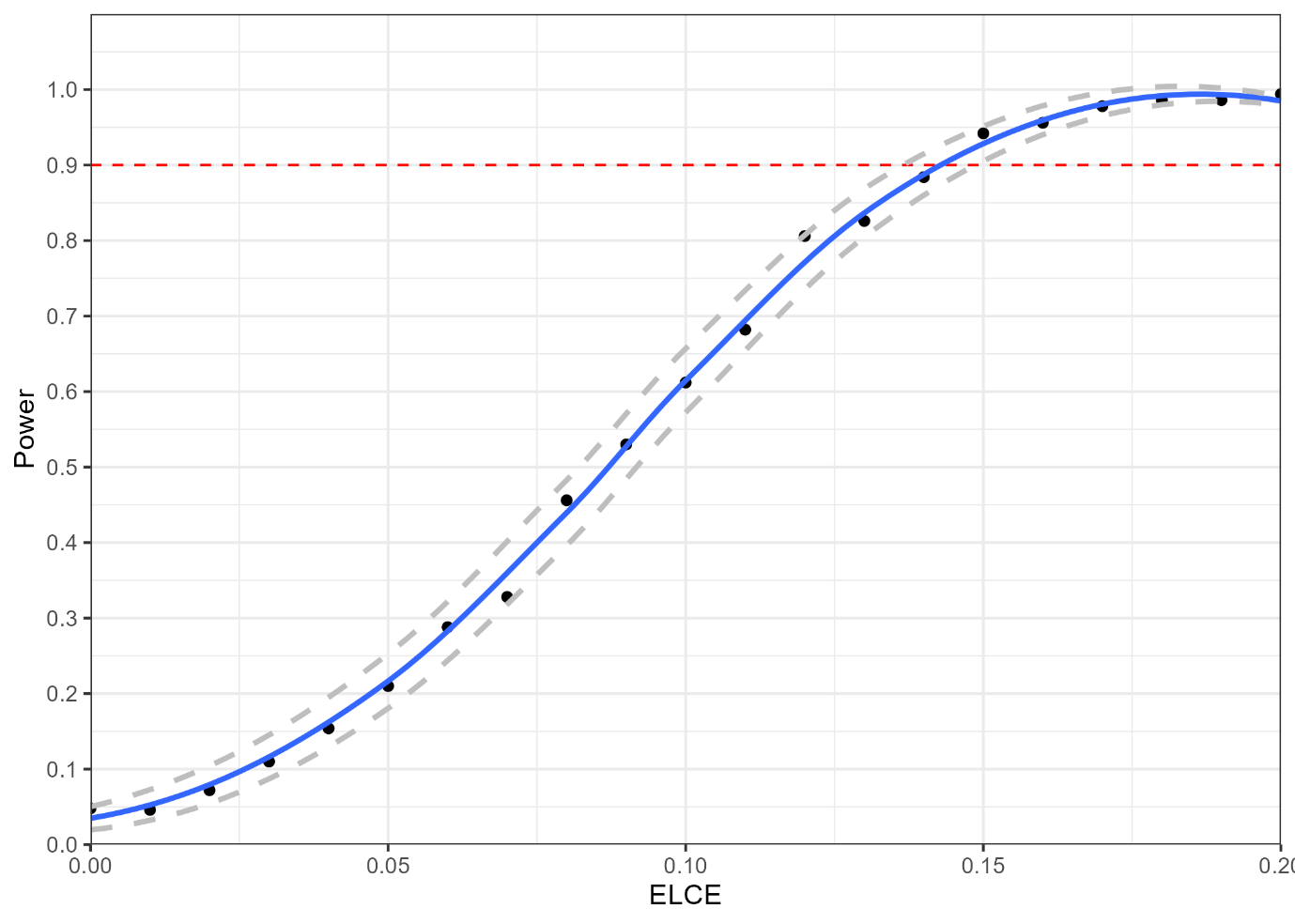


*Note.* Statistical power in logistic regression for *n* = 5009 (proportion of children with comprehension weaknesses = 0.18), with one centered continuous predictor (language ability) fixed at -0.695 and a second (ELCE) of varying effect sizes. Grey dashed lines mark 95% confidence intervals. Power is estimated from 500 simulated datasets; the red dashed line marks the desired power threshold.

### Figure S3. Statistical power for predicting the probability of group outcomes based on an interaction between two continuous predictors


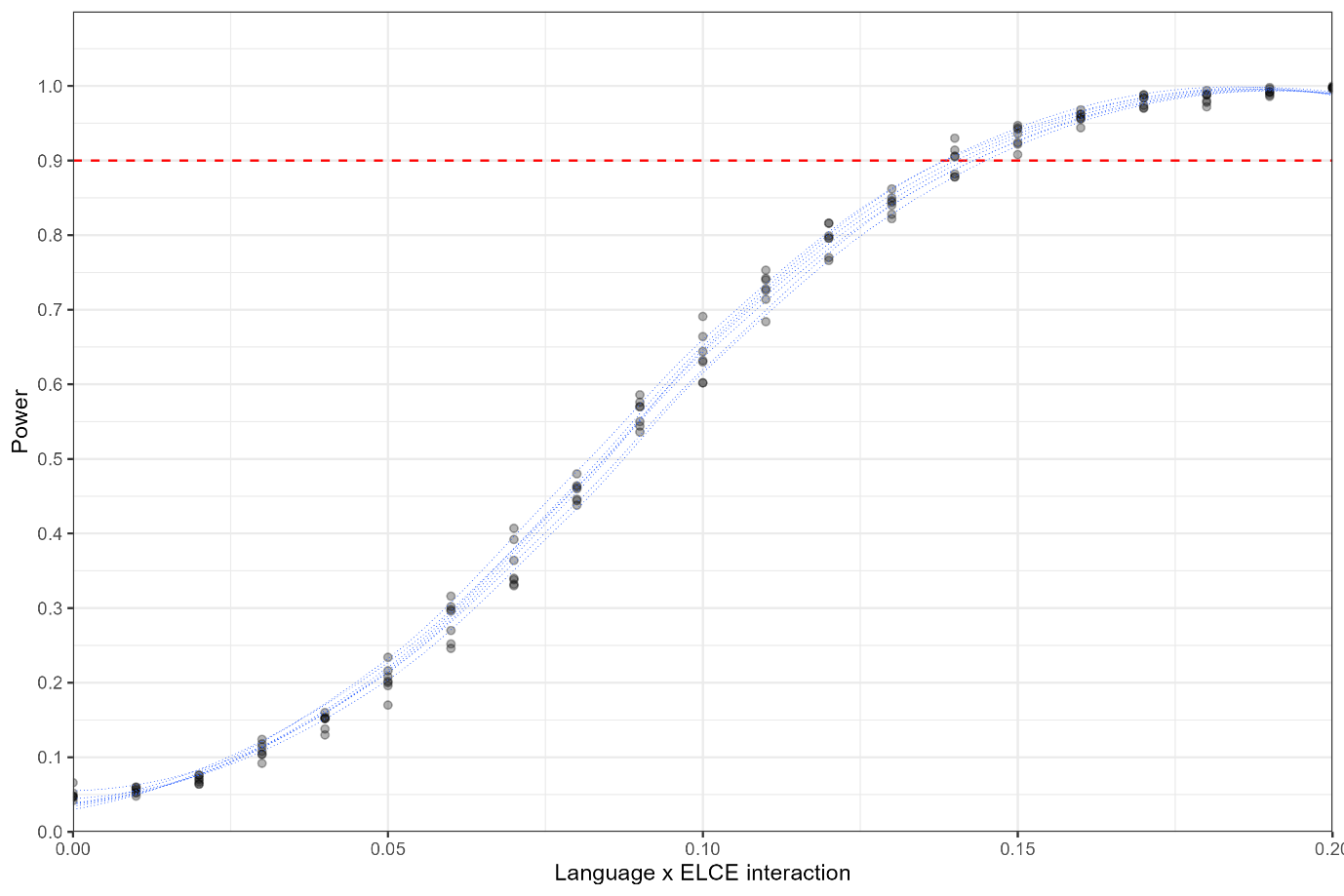


*Note.* Statistical power in logistic regression for *n* = 5009 (proportion of children with comprehension weaknesses = 0.18). One centered continuous predictor (language ability) is fixed at -0.695, and each line represents the power to detect the interaction at a different effect size of ELCE (ranging from -0.2 to -0.5, under the assumption that the main effect will be larger than the interaction). Power is estimated from 500 simulated datasets; the red dashed line marks the desired power threshold.

### Figure S4. Statistical power for predicting the probability of group outcomes based on a dichotomous risk predictor


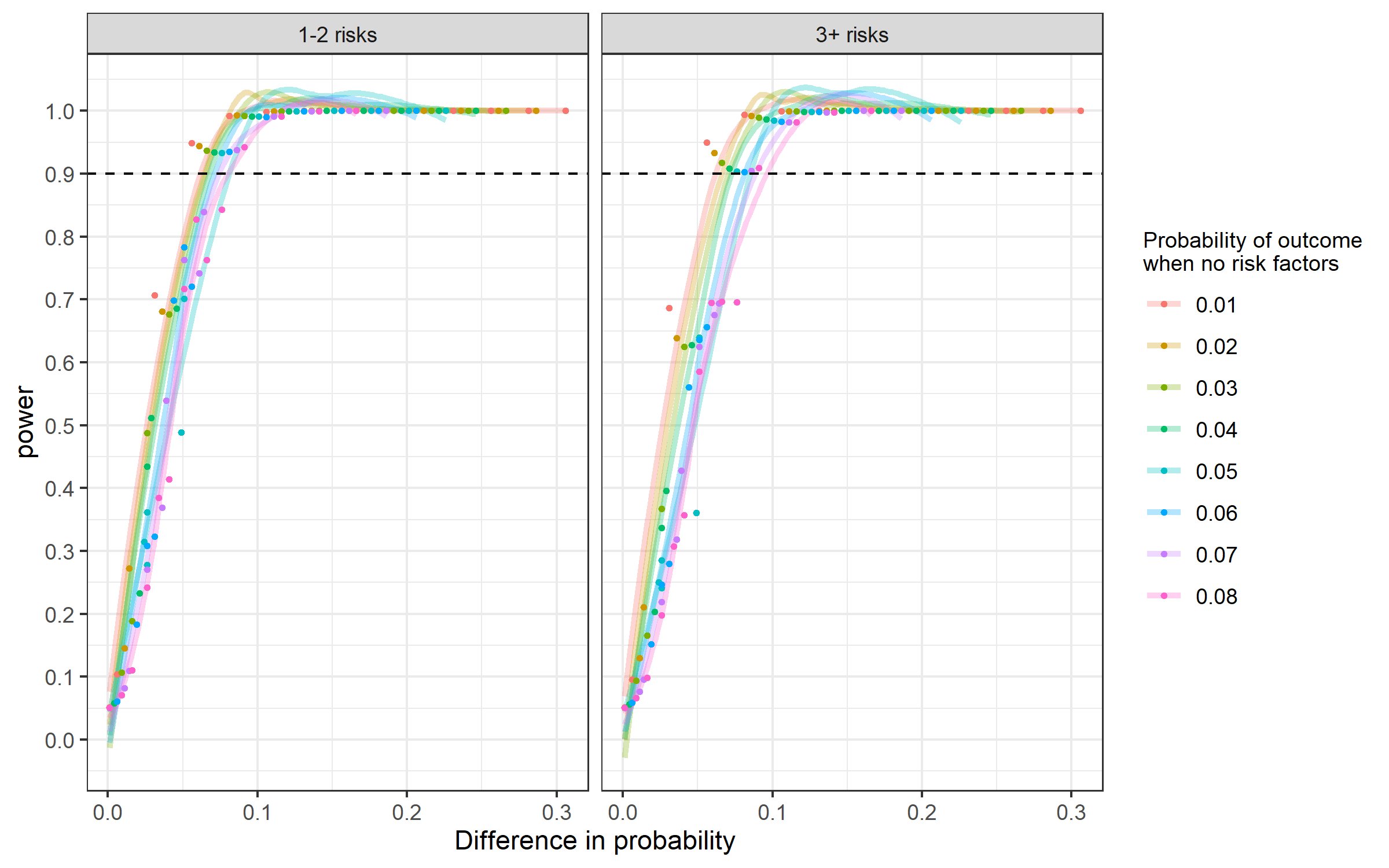


*Note.* Statistical power in logistic regression for *n* = 687 participants, plotted for different probabilities of [*y* = 1] for [risks = 0] (colour), with the difference in probability between [risks = 0] and [risks > 0] along the x-axis. The two panels represent different Bernoulli distributions for the risk predictor, based on the prevalence of 1-2 risks (35%) and 3+ risks (24%; Hayiou-Thomas et al., 2021). Dashed black lines mark the 90% power threshold.

### Figure S5. Statistical power for detecting the difference in AUC between two ROC curves


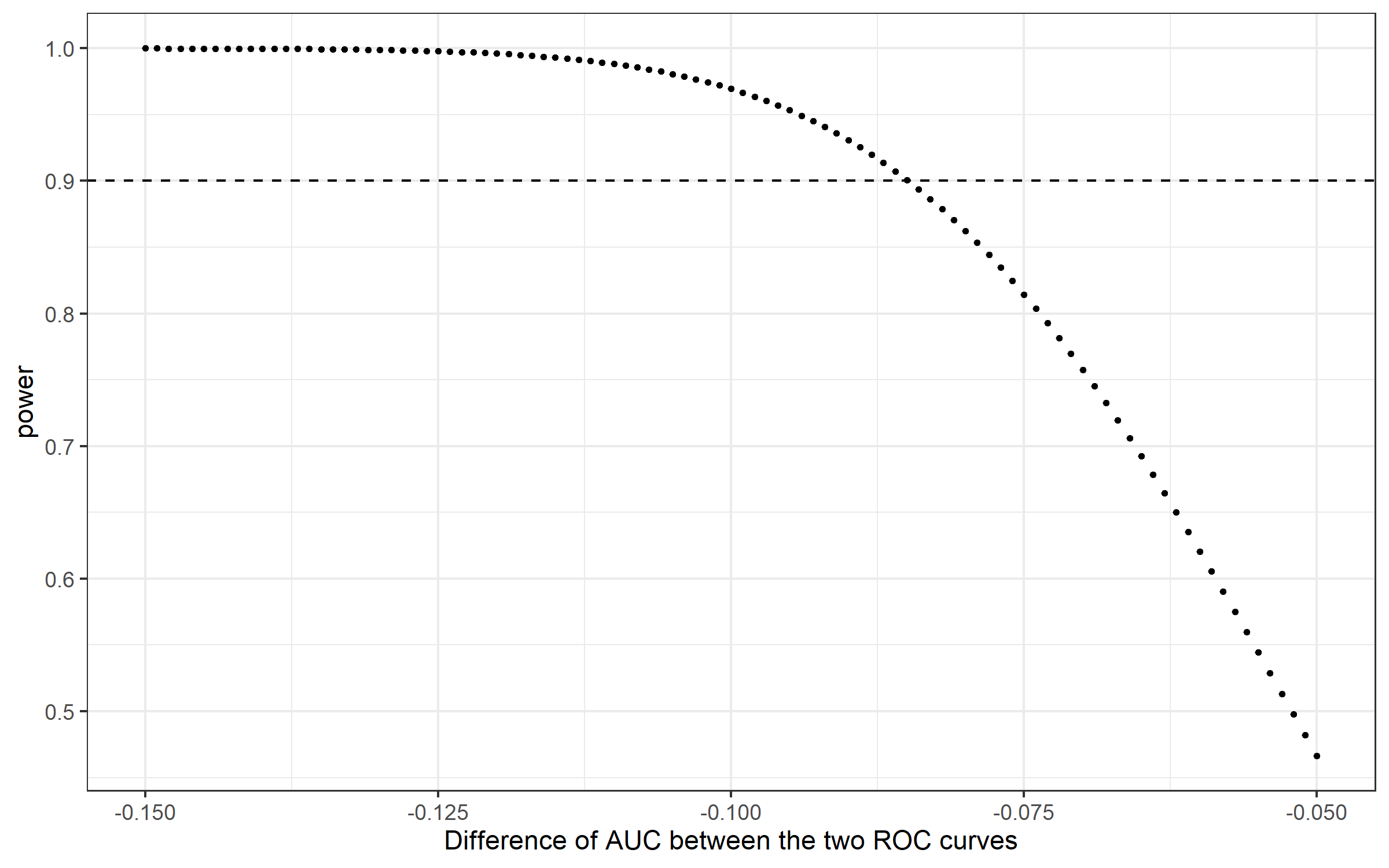


*Note.* Statistical power for sample of 125 cases (children with comprehension weaknesses) and 562 controls, with the ROC curves estimated through data simulation from parameters reported in Hayiou-Thomas et al. (2021). The dashed black line marks the 90% power threshold.

### Table S1. Complete case analysis for differences in language scores at 15, 24, and 36 months

|  | Typically developing readers  *M (SD)* | Children with comprehension weaknesses  *M (SD)* | *t* | *df* | *p* | *d* |
| --- | --- | --- | --- | --- | --- | --- |
| 15 m | 0.02 (0.89) | -0.02 (0.93) | 1.14 | 1207.2 | .253 | 0.04 |
| 24 m | 0.07 (0.91) | 0.02 (0.96) | 1.27 | 1031.4 | .205 | 0.05 |
| 38 m | 0.07 (0.74) | -0.03 (0.82) | 3.09 | 1064.3 | .002 | 0.13 |

### Table S2. Complete case analysis for predicting reading outcomes on the basis of preschool language and environmental measures

|  | Model 1: Language only | | | | Model 2: Language & environment | | | |
| --- | --- | --- | --- | --- | --- | --- | --- | --- |
|  | *β* | *SE* | *Z* | *p* | *β* | *SE* | *Z* | *p* |
| (Intercept) | -1.56 | 0.04 | -39.17 | < .001 | -1.55 | 0.04 | -37.84 | < .001 |
| Language (38m) | -0.16 | 0.05 | -3.34 | .001 | -0.17 | 0.05 | -3.26 | .001 |
| ELCE | - | - | - | - | -0.02 | 0.07 | -0.23 | .819 |
| Language X ELCE | - | - | - | - | -0.05 | 0.07 | -0.70 | .484 |
| *Note.* As in the main analysis, model fit was poor across both Model 1 (R2 = .003) and Model 2 (R2 = .004) and neither could predict reading group outcomes. There was no difference in fit between the two models (*p* = .778). | | | | | | | | |

### Table S3. Classification performance when using early language ability to predict comprehension group outcomes (complete case analysis)

| Threshold justification | Probability threshold | Predictive accuracy | Sensitivity | Specificity | Positive predictive value | Negative predictive value |
| --- | --- | --- | --- | --- | --- | --- |
| ROC optimal | 0.17 | 0.56 | 0.49 | 0.57 | 0.2 | 0.84 |
| 90% sensitivity | 0.16 | 0.25 | 0.9 | 0.11 | 0.18 | 0.84 |

### Table S4. Final CFA for Study 2

| term | estimate | std.error | statistic | p.value | std.all |
| --- | --- | --- | --- | --- | --- |
| **Latent variables**  Language =~  mtcq_say_ageZ_kg | 0.52 | 0.04 | 13.96 | 0 | 0.52 |
| mtcq_comb_ageZ_kg | 0.49 | 0.04 | 12.56 | 0 | 0.48 |
| wppsi_vcb_std_cif3 | 1.7 | 0.11 | 15.32 | 0 | 0.59 |
| bus_inf_ageZ_cif4 | 0.4 | 0.04 | 9.4 | 0 | 0.4 |
| bus_snt_ageZ_cif4 | 0.45 | 0.05 | 9.36 | 0 | 0.4 |
| mtcq_und_ageZ_kg | 0.27 | 0.04 | 6.84 | 0 | 0.27 |
| rdls_std_cif2 | 0.39 | 0.03 | 11.28 | 0 | 0.53 |
| rdls_std_cif4 | 0.16 | 0.02 | 8.42 | 0 | 0.42 |
| wppsi_comp_std_cif3  Nonverbal IQ =~ | 2.08 | 0.1 | 20.85 | 0 | 0.76 |
| wppsi_bloc_std_cif3 | 2.25 | 0.1 | 21.91 | 0 | 0.76 |
| wppsi_geom_std_cif3 | 1.68 | 0.09 | 19.33 | 0 | 0.68 |
| wppsi_maze_std_cif3 | 1.78 | 0.11 | 16.44 | 0 | 0.6 |
| wppsi_obja_std_cif3 | 1.64 | 0.12 | 14.11 | 0 | 0.53 |
| wppsi_pcmp_std_cif3 | 1.53 | 0.09 | 16.14 | 0 | 0.59 |
| Verbal short-term memory =~  ctnr_ageZ_cif4 | 0.54 | 0.04 | 13.49 | 0 | 0.54 |
| digsp_ageZ_cif3 | 0.82 | 0.04 | 19.38 | 0 | 0.81 |
| digsp_ageZ_cif4 | 0.72 | 0.04 | 18.98 | 0 | 0.72 |
| ELCE =~  elce_mcdt_sum | 0.68 | 0.06 | 11.53 | 0 | 0.45 |
| elce_mca_sum | 1.96 | 0.11 | 17.26 | 0 | 0.67 |
| elce_int_sum | 1.46 | 0.07 | 19.49 | 0 | 0.75 |
| elce_res_sum | 0.61 | 0.08 | 7.3 | 0 | 0.29 |
| elce_oth_sum | 0.72 | 0.08 | 9.46 | 0 | 0.38 |
| Phonemic awareness =~  icdt_ageZ_cif4 | 1 | 0.03 | 39.08 | 0 | 1 |
| **Covariances**  lang ~~ phon | 0.53 | 0.03 | 16.19 | 0 | 0.53 |
| nonverbal ~~ phon | 0.47 | 0.03 | 13.93 | 0 | 0.47 |
| stm ~~ phon | 0.43 | 0.04 | 11.87 | 0 | 0.43 |
| elce ~~ phon | 0.27 | 0.04 | 6.62 | 0 | 0.27 |
| lang ~~ nonverbal | 0.68 | 0.03 | 20.99 | 0 | 0.68 |
| lang ~~ stm | 0.57 | 0.04 | 14.32 | 0 | 0.57 |
| lang ~~ elce | 0.42 | 0.04 | 9.92 | 0 | 0.42 |
| nonverbal ~~ stm | 0.42 | 0.04 | 10.15 | 0 | 0.42 |
| nonverbal ~~ elce | 0.28 | 0.05 | 6.15 | 0 | 0.28 |
| stm ~~ elce | 0.19 | 0.05 | 4.05 | 0 | 0.19 |
|  |  |  |  |  |  |
|  |  |  |  |  |  |
| bus_inf_ageZ_cif4 ~~ bus_snt_ageZ_cif4 | 0.73 | 0.05 | 14.14 | 0 | 0.79 |
| mtcq_say_ageZ_kg ~~ mtcq_und_ageZ_kg | 0.29 | 0.03 | 8.86 | 0 | 0.36 |
| **Variances**  icdt_ageZ_cif4 | 0 | 0 | NA | NA | 0 |
| mtcq_say_ageZ_kg | 0.73 | 0.04 | 17.79 | 0 | 0.73 |
| mtcq_comb_ageZ_kg | 0.79 | 0.04 | 17.99 | 0 | 0.77 |
| wppsi_vcb_std_cif3 | 5.5 | 0.33 | 16.67 | 0 | 0.66 |
| bus_inf_ageZ_cif4 | 0.84 | 0.05 | 17.11 | 0 | 0.84 |
| bus_snt_ageZ_cif4 | 1.01 | 0.07 | 15.31 | 0 | 0.84 |
| mtcq_und_ageZ_kg | 0.93 | 0.05 | 19.78 | 0 | 0.93 |
| rdls_std_cif2 | 0.4 | 0.02 | 16.01 | 0 | 0.72 |
| rdls_std_cif4 | 0.12 | 0.01 | 16.47 | 0 | 0.82 |
| wppsi_comp_std_cif3 | 3.23 | 0.26 | 12.45 | 0 | 0.43 |
| wppsi_bloc_std_cif3 | 3.66 | 0.28 | 13.04 | 0 | 0.42 |
| wppsi_geom_std_cif3 | 3.2 | 0.21 | 15.5 | 0 | 0.53 |
| wppsi_maze_std_cif3 | 5.61 | 0.33 | 16.92 | 0 | 0.64 |
| wppsi_obja_std_cif3 | 7.01 | 0.39 | 17.83 | 0 | 0.72 |
| wppsi_pcmp_std_cif3 | 4.28 | 0.25 | 17.02 | 0 | 0.65 |
| ctnr_ageZ_cif4 | 0.71 | 0.04 | 16.38 | 0 | 0.71 |
| digsp_ageZ_cif3 | 0.36 | 0.04 | 8.01 | 0 | 0.35 |
| digsp_ageZ_cif4 | 0.48 | 0.04 | 12.18 | 0 | 0.48 |
| elce_mcdt_sum | 1.83 | 0.1 | 18.35 | 0 | 0.8 |
| elce_mca_sum | 4.77 | 0.36 | 13.4 | 0 | 0.55 |
| elce_int_sum | 1.63 | 0.16 | 9.94 | 0 | 0.43 |
| elce_res_sum | 3.95 | 0.2 | 19.63 | 0 | 0.91 |
| elce_oth_sum | 3.11 | 0.16 | 18.97 | 0 | 0.86 |
| lang | 1 | 0 | - | - | 1 |
| nonverbal | 1 | 0 | - | - | 1 |
| stm | 1 | 0 | - | - | 1 |
| elce | 1 | 0 | - | - | 1 |
| phon | 1 | 0 | - | - | 1 |
|  |  |  |  |  |  |
| **Intercepts**  mtcq_say_ageZ_kg ~1 | 0 | 0.03 | -0.14 | 0.89 | 0 |
| mtcq_comb_ageZ_kg ~1 | -0.05 | 0.04 | -1.4 | 0.16 | -0.05 |
| wppsi_vcb_std_cif3 ~1 | 9.98 | 0.1 | 96.3 | 0 | 3.45 |
| bus_inf_ageZ_cif4 ~1 | -0.02 | 0.04 | -0.64 | 0.52 | -0.02 |
| bus_snt_ageZ_cif4 ~1 | -0.16 | 0.04 | -3.71 | 0 | -0.15 |
| mtcq_und_ageZ_kg ~1 | 0 | 0.03 | -0.08 | 0.94 | 0 |
| rdls_std_cif2 ~1 | 1.09 | 0.03 | 37.32 | 0 | 1.47 |
| rdls_std_cif4 ~1 | 0.59 | 0.02 | 37.25 | 0 | 1.52 |
| wppsi_comp_std_cif3 ~1 | 10.16 | 0.1 | 103.93 | 0 | 3.69 |
| wppsi_bloc_std_cif3 ~1 | 11.55 | 0.11 | 109.24 | 0 | 3.91 |
| wppsi_geom_std_cif3 ~1 | 10.24 | 0.09 | 116.64 | 0 | 4.18 |
| wppsi_maze_std_cif3 ~1 | 10.01 | 0.11 | 94.06 | 0 | 3.38 |
| wppsi_obja_std_cif3 ~1 | 11.53 | 0.11 | 103.14 | 0 | 3.7 |
| wppsi_pcmp_std_cif3 ~1 | 12.83 | 0.09 | 138.85 | 0 | 4.99 |
| ctnr_ageZ_cif4 ~1 | -0.02 | 0.04 | -0.5 | 0.61 | -0.02 |
| digsp_ageZ_cif3 ~1 | -0.03 | 0.04 | -0.77 | 0.44 | -0.03 |
| digsp_ageZ_cif4 ~1 | -0.01 | 0.04 | -0.28 | 0.78 | -0.01 |
| elce_mcdt_sum ~1 | 8.11 | 0.05 | 155.92 | 0 | 5.35 |
| elce_mca_sum ~1 | 32.31 | 0.1 | 318.57 | 0 | 11 |
| elce_int_sum ~1 | 28.1 | 0.07 | 419.81 | 0 | 14.51 |
| elce_res_sum ~1 | 21.49 | 0.07 | 298.48 | 0 | 10.34 |
| elce_oth_sum ~1 | 8.22 | 0.07 | 124.42 | 0 | 4.32 |
| icdt_ageZ_cif4 ~1 | -0.01 | 0.04 | -0.35 | 0.73 | -0.01 |
| lang ~1 | 0 | 0 | - | - | 0 |
| nonverbal ~1 | 0 | 0 | - | - | 0 |
| stm ~1 | 0 | 0 | - | - | 0 |
| elce ~1 | 0 | 0 | - | - | 0 |
| phon ~1 | 0 | 0 | - | - | 0 |
